# Protein hydrolysates produced from rock lobster (*Jasus edwardsii*) Head: emulsifying capacity and food safety

**DOI:** 10.1002/fsn3.352

**Published:** 2016-03-10

**Authors:** Shan He, Trung T. Nguyen, Peng Su, Wei Zhang

**Affiliations:** ^1^ Centre for Marine Bioproducts DevelopmentFlinders UniversityAdelaideSA5042Australia; ^2^ Department of Medical BiotechnologySchool of MedicineFlinders UniversityAdelaideSA5042Australia; ^3^ Present address: Medical BiotechnologyFlinders UniversityLevel 4 Health Science Building, Bedford Park, 5042AdelaideSouth AustraliaAustralia

**Keywords:** Food safety, lobster head, lobster protein hydrolysates, process development

## Abstract

Lobster protein hydrolysates (LPH) were produced by an enzymatic process using a proteinase Alcalase, and a chemical process at strong alkaline condition (pH of 14), from rock lobster head (RLH), respectively. The chemical process recovered about 30% more protein than the enzymatic process (84.9% recovery of total protein in RLH by the chemical process and 54.5% recovery of total protein in RLH by the enzymatic process). The emulsifying capacity of LPH produced by the chemical process (69.7 m^2^/g) was significantly higher than the emulsifying capacity of the LPH produced by the enzymatic process (20.7 m^2^/g), and also exceeds the emulsifying capacity of cow gelatine (50.3 m^2^/g), a commercial emulsifier in the food industry. LPH produced by the chemical process possess 30.3% essential amino acids. This content is comparable with the essential amino acid content of fish protein, a commonly recognized food resource for essential amino acid supplement for human. The content of heavy metals, including inorganic arsenic, of LPH is lower than the standard levels regulated by Food Standard Australia and New Zealand (FSANZ). These results demonstrated the potential value of LPH used as a safe emulsifier with significant nutritional value for the food industry.

## Introduction

Lobster heads consist of material left over from the tail meat, a consumable part of lobsters. The percentage of lobster heads is more than 50% of the whole lobster by weight (Bechtel 2003), which imposes a disposal cost in the absence of value‐added solutions. The high percentage of fish processing coproducts is not unique for lobster but common for all other seafood. In Australia, seafood industries discard over 100,000 tons of various marine coproducts annually (Peter and Clive 2006). Currently, it costs Australian dollar (AUD) 150 per ton to discard these coproducts because they are classified as certified waste due to their high content of organic matter (Oliveira et al. 2009). As the largest producer of rock lobster in the world (Tsvetnenko et al. 1996), Australia produces about 3,000 tons of rock lobster heads (RLH) annually. By estimation, the Australian lobster industry could spend over AUD 500,000 per annum on the disposal of RLH, rather than generating benefits via productive utilization and value adding. This inefficient business model has been identified to not only be cost‐ineffective but also environmentally unfriendly. Therefore, the utilization of seafood coproducts, including RLH, to produce value‐added products has been highlighted by the Australian Seafood Industry Council as a high priority area that needs to be addressed (Ian et al. 2004).

Though RLH are not directly consumable, they contain many valuable such as protein, chitin, and omega‐3 fatty acids (Trung et al.^2015^). While there is no report on how RLH could be used for value‐added products. The possible solution can be learned from the comprehensive studies on the processes developed for value adding to fish processing coproducts. The recovery of fish protein hydrolysates appears to be one of the valuable products for the seafood industry. Fish protein hydrolysates are fish proteins that are broken down into peptides of various sizes (He et al. 2013). Fish protein hydrolysates have been produced from fish processing coproducts of many fish species, such as Shark (Diniz and Martin 1997), Salmon (Gbogouri et al. 2004), Blue whiting (Geirsdottir et al. 2011), and Tilapia (Poh et al. 1997). They were produced using either chemical or enzymatic processes (He et al. 2013). The advantages and drawbacks of these two processes has been compared (He et al. 2015). It is reasonable to believe that these two processes can also be applied to extract lobster protein hydrolysates (LPH) from RLH.

In general, fish protein hydrolysates have improved physicochemical properties, such as oil‐binding capacity and emulsifying capacity, compared with intact fish protein. These improved properties enable fish protein hydrolysates to be used as functional food ingredients in many food products, such as meat products and spread‐texture food. He et al. (2012) found that the emulsifying capacity (53.43 m^2^/g) of fish protein hydrolysates produced from the processing coproducts of Yellowtail kingfish exceeds that of cow gelatine (50.30 m^2^/g), the standard emulsifier commonly used in the food industry. Diniz and Martin (1997) demonstrated that the emulsifying capacity of fish protein hydrolysates produced from processing coproducts of Shark (55 mL/0.5 g sample) is higher than the emulsifying capacity of intact Shark protein (39 mL/0.5 g sample). Slizyte et al. (2005) showed that the oil‐binding capacity of fish protein hydrolysates produced from processing coproducts of Cod (5.0 g oil/g protein) is higher than that of soy protein (1.2 g oil/g protein), the standard oil binder commonly used in the food industry. In this study, the physicochemical properties of LPH produced from RLH were tested to understand their potential applications.

To apply the LPH as food ingredients into food formulations, food safety is a primary concern. The most serious food safety concern for seafood products is heavy metal content, especially the content of Arsenic that is known to cause cancer, and many other serious health problems (American Cancer Society, 2014). It was shown that common seafood, such as fish and lobster, may contain arsenic at exceedingly high concentrations (Kevin 2010). About 90% of the arsenic in US diets comes from seafood (Jonathan and Dean 2007). Therefore, it is crucial to know the content of heavy metals, especially arsenic, in food ingredients made from seafood, such as LPH.

The objective of this study was therefore to develop a cost‐effective process to produce LPH from RLH, and characterize its physicochemical properties for potential applications. This study also comprehensively analyzed the content of heavy metals, especially arsenic, of RLH and LPH to meet the food safety regulation.

## Materials and Methods

### Materials

RLH were supplied by Ferguson Australia (16 Circuit Drive, Hendon, South Australia, Australia). All chemicals used in this study were purchased from Sigma‐Aldrich Australia Pty. Ltd (Castile Hill, Australia). Commercial Alcalase was provided by Novozymes Australia Pty. Ltd (North Rocks, Australia).

### Sample collection and preparation

Each 10 kg of RLH provided by Ferguson Australia was packed in a sealed plastic bag, and transported to the cold room (−74°C) at the Department of Medical Biotechnology, Flinders University. They were stored there until use. The RLH was thawed under the running tap water for about 30 min, and minced using heavy‐duty mincer immediately before use.

### Chemical composition analysis

Water and fat contents were determined using AOAC methods 950.46 and 960.39 (AOAC, 2000), respectively. For protein content of minced RLH, the RLH was fully mixed with the lysis buffer (2% w/v Sodium dodecyl sulfate (SDS), 0.1mol/L Dithiothreitol (DTT), 60 mmol/L Tris HCl, pH 7.5) at a 1:10 (w/v) ratio. Protein from RLH was extracted using the lysis buffer for 15 h at rooming temperature of 25°C. The protein content of the lysis buffer extract was measured using the BCA method (Product code B9643, Sigma). Then the protein content of the lysis buffer extract was converted into protein content of minced RLH using the measured amount of RLH and measured volume of lysis buffer. All experiments of this study were carried out in a laboratory with constant room temperature.

### Process development

#### Enzymatic process

Frozen minced RLH were thawed for about 30 min in running tap water before use. The enzymatic process was performed using the commercial protease, Alcalase, in mince‐water slurries. The mince‐water slurries were made by mixing evenly 50 g of tap water with 50 g of minced RLH. The temperature of mince‐water slurries was adjusted to 55°C to meet the optimal temperature of Alcalase. The pH of the mince‐water slurries was adjusted to 8.0, the optimal pH of Alcalase. Alcalase was chosen for this study because the preliminary tests of three most commonly used proteases of Flavourzyme, Neutrase, and Alcalase, indicated the highest production yield achieved by Alcalase. Enzymatic processing was undertaken in a water‐bath. The processing time was set at 1 h because the lobster industry prefer to develop LPH production processes in a short time of 1 h, rather than a long time of about 3 h used in other enzymatic processes for fish processing wastes (Klompong et al. 2007; Wasswa et al. 2007). The enzyme: substrate (E:S) ratio was set at 7% (w/w), because the results of preliminary trials showed that the production yield achieved by E:S of 7% was significantly higher than that by E:S of 6%, whereas E:S of 7% has reached the optimum E:S ratio. Higher E:S ratio did not make the production yield significantly different. Substrate refers to the weight of minced RLH in this study.

Therefore, the optimum condition of enzymatic process was chosen as using Alcalase with E:S ratio of 7% for 1 h. Only the results from the optimum enzymatic process, were shown in this study for clearer comparison.

#### Chemical process

Frozen minced RLH were thawed for about 30 min in running tap water before use. Mince‐water slurries, made by mixing evenly 50 g of tap water with 50 g of minced RLH, were adjusted to pH 14 using 2.5 mol/L NaOH solution. Chemical process was undertaken in an Autoclave (Atherton, Australia) at a temperature of 121°C and 15‐psi pressure for 20 min, the standard processing time for this type of chemical process as reported in previous studies (Aaslying et al. 1998; Kristinsson and Rasco 2000).

#### Separation of LPH liquid after processing

Processed mince‐water slurries from the aforementioned enzymatic process and chemical process were centrifuged at 4000*g* for 30 min, resulting in three layers: a lipid layer on the top, protein hydrolysate solution in the middle and a semisolid layer at the bottom. The lipid layer was removed by aspiration, then the protein hydrolysate solution was decanted, collected and freeze dried. The freeze‐dried protein hydrolysates were weighed to record yields then placed in 50‐mL‐sealed tubes and stored in desiccators at room temperature until use.

### Physicochemical properties tests

The oil‐binding capacity was measured using a published method (Wasswa et al. 2007) with slight modifications. Half a gram of each LPH powder was added to 9 g of canola oil in a 50 mL centrifuge tube, mixed for 1 min with a vortex mixer, then centrifuged at 2000*g* for 30 min at room temperature. The weight of the oil separated from the LPH was measured and the oil‐binding capacity was calculated as the amount of oil absorbed per 1 g sample. Egg white powder (self‐produced by freeze‐drying egg white), a commonly used oil binder, was used as reference.

The emulsifying capacity was measured using a published method (Klompong et al. 2007) with slight modifications. Three grams of canola oil and 10 mL 1% (w/v) solution of each LPH powder sample in water was homogenized in a bio‐homogenizer (IKA Labortechnik, Staufen, Germany) at a speed of 25,000 rpm for 1 min. The solvent of the solution was water. The pH of each test sample solution was adjusted to 7.0 before adding canola oil. 50 *μ*L of emulsion was pipetted from the bottom and then mixed with 5 mL of 0.1% (w/v) sodium dodecyl sulfate (SDS) solution. The absorbance (A500) of the SDS‐diluted solution was measured immediately at 500 nm by a UV–Vis spectrophotometer (100 Tigan Street, Winooski, VT). Emulsifying capacity was calculated as follows:

$$\text{Emulsifying capacity}\left(m^{2}/g\right)\;=\;\frac{2\times 2.303\times A500}{0.25\times \text{protein weight(g)}}$$

Cow gelatine powder, the commercial emulsifying agent, was used as a reference.

### Amino acid profile of LPH

The amino acid profile of LPH was determined following the methods of Shahidi et al. (1995) with slight modifications. LPH were hydrolyzed with 6mol/L HCl at 100°C for 25 h. The acid was then removed under vacuum and the resultant dried material was reconstituted with a pH 2.2 lithium citrate buffer. The amino acid composition was quantified using a high‐resolution RP‐ HPLC column on an ultra performance liquid chromatography (UPLC) system. This instrument consists of an ACQUITY UPLC system with UV detector from Waters Corporation (Milford, MA). The wavelength of the UV detector was set at 260 nm. For all analyses, a Waters AccQ‐Tag Ultra column (BEH C18, 1.7 *μ*m; 2.1 × 100 mm) was used with the column temperature at 55°C, and a solvent flow rate of 0.7 mL/min. The sample concentration was 50 pmol on column and the injection volume was 0.5 *μ*L. AccQ‐Tag Ultra Eluent A and AccQ‐Tag Ultra Eluent B (Water Corporation, Milford, MA) were applied as Mobile phase A (10 mmol/L ammonium formate in 90/10 (v/v) water/methanol with 0.3% (w/w) formic acid) and Mobile phase B (10 mmol/L ammonium formate with 0.5% (w/w) formic acid in MeOH), respectively.

### Heavy metal content of RLH and LPH

The heavy metal content was determined by inductively coupled plasma‐mass spectrometry and inductively coupled atomic emission spectrometry using standard United States Environmental Protection Agency (USEPA) methods 6010B and 6020 (USEPA, 1996).

### Statistical analysis

Measurements of production yield, oil‐binding capacity, emulsifying capacity, water content, and fat content were performed in triplicate. Data were presented as the mean with standard deviation, and subjected to one‐way analysis of variance (ANOVA) and least significant difference (LSD) using MINITAB Statistical Software (1829 Pine Hall Road, State College, PA, USA) v15. The significance was judged statistically by the *F* value at a probability (*P*) below 0.05.

## Results and Discussion

### Chemical composition of RLH

The water, protein, and fat contents of RLH are shown in Table 1. The protein content of RLH is about 12.7% based on its wet weight. This figure is in agreement with the protein content of many fish processing coproducts. Fish processing coproducts are used for comparison because they have been identified as a good resource for the production of protein hydrolysate (Kristinsson and Rasco 2000; Sanmartin et al. 2009). He et al. (2011) reported that the protein content of the head and frame of Yellowtail kingfish, one major commercial fish species in Australia, is about 14.76% and 13.88%, respectively, based on wet weight. Sathivel et al. (2004) demonstrated that the protein content of the head and frame of Atlantic herring is about 13.1% and 16.9%, respectively, based on wet weight. The fish processing coproducts of these fish species have been used to produce fish protein hydrolysates, due to their high protein content (Sathivel et al. 2004; He et al. 2012). Therefore, based on the protein content, RLH can be a good source for the production of LPH.

**Table 1:** Water, protein, fat, and chitin content of rock lobster head

	Water content^a^ (%, (w/w))	Protein content^a^ (%, (w/w))	Fat content^a^ (%, (w/w))
Rock lobster head	70.8 ± 3.56	12.7 ± 1.89	0.1 ± 0.03

a Average of the reading of three samples per trial ± standard deviation of the mean.

Furthermore, when the fat content of raw materials is considered, RLH can be regarded as a better raw material for the production of protein hydrolysates, compared with fish processing coproducts. The process of using fish processing coproducts as raw material to produce fish protein hydrolysates has been comprehensively studied (Shahidi et al. 1995; Gbogouri et al. 2004; Sarmadiadi and Ismail 2010). One costly step of this process is defatting. This step is necessary because of the high fat content of fish processing coproducts. He et al. (2011) showed that the fat content of fish processing coproducts from different fish species is in the range 20–30%, whereas, it can be seen from Table 1 that the fat content of RLH is only about 0.09%. Therefore, in industrial production, the step of defatting could cost much less, or is probably removed, if using RLH as raw material to produce LPH. This comparison shows that it may be cheaper for the production of lobster protein hydrolysates, in comparison with fish processing coproducts.

### Production yield and protein recovery of LPH by the chemical and enzymatic processes

Production yields of LPH, the crucial data for industrial production, are shown in Table 2. Protein recovery in Table 2 was calculated as the percentage of production yield in the total protein of raw material (as mentioned in section 2.4 processing methods, 50 g minced RLH was used as raw material in this study; as mentioned in Table 1, the protein content of RLH is 12.7%. Therefore, the total protein of raw material is 50 g × 12.70% = 6.35 g).

**Table 2:** Production yield and protein recovery of lobster protein hydrolysates produced from chemical and enzymatic processes

	Production yield^a^ (g/50 g fresh rock lobster head)	Protein recovery^a^ (% (w/w))
Chemical process	5.4^a ^± 0.39	84.9^a ^± 5.91
Enzymatic process	3.5^b ^± 0.43	54.5^b ^± 6.54

a Average of the reading of three samples per trial ± standard deviation of the mean.

value within the same column bearing different letters are significantly different at *P* < 0.05, letters do not apply to any row.

Chemical and enzymatic processes used in this study are the two most common processes for the production of protein hydrolysates (Kristinsson and Rasco 2000). Though many studies encourage the application of the enzymatic process for industrial production of protein hydrolysates, due to its merits of producing protein hydrolysates with higher nutritive value, more homogeneous molecular weights and less bitterness (He et al. 2013), this process has only applied on a laboratory scale so far, due to its drawbacks of long process time, low production yield, and extra enzyme cost. On the other hand, though the chemical process is considered as obsolete for protein hydrolysates production by many studies based on laboratory scale (Kristinsson and Rasco 2000; Sanmartin et al. 2009), its advantages, such as low cost, short processing time, and high production yield have been commonly recognized by industrial production. Therefore, a comparison of the enzymatic and chemical processes for the production of lobster protein hydrolysates from RLH is necessary.

The typical processing time of an enzymatic process is up to 3 h (He et al. 2015). The food industry proposed that this processing time is unacceptable for industrial production on daily basis; it needs to be shortened to 1 h. Therefore, the processing time of 1 h was applied in this study. Table 2 shows that the production yield of the enzymatic process is 3.46 g, with a protein recovery of 54.49%. This protein recovery is comparable with the protein recovery of other protein hydrolysates, such as fish protein hydrolysates produced from Atlantic salmon (50.34%) and Yellowtail kingfish (69.19%), with similar processing conditions (He et al. 2012); whereas the production yield (3.46 g) and protein recovery (52%) of the enzymatic process is much lower than the production yield (5.39 g) and protein recovery (84.88%) of the chemical process in this study (Table 2). The processing time of the chemical process is only 20 min, much shorter than the processing time of the enzymatic process (1 h). A similar trend was also found in the production of fish protein hydrolysates using processing coproducts of Yellowtail kingfish as raw material: the chemical process provided a protein recovery of 86.53%, which is higher than the protein recovery using the enzymatic process (41.94%), with the same processing time of 20 min. These results demonstrated the advantage of the chemical process in protein recovery and production yield, in comparison with the enzymatic process.

Table 2 clearly shows that the chemical process provides significantly higher production yield and protein recovery. However, according to previous studies, the chemical process results in poor nutritive values of protein hydrolysates (Theodore and Kristinsson 2007). Due to this, protein hydrolysates produced from the chemical process are usually used for low‐value products such as fertilizer, with a profit of only US 50 cent/ton, or as a nitrogen source for the growth of lactic acid bacteria (Kristinsson and Rasco 2000).

### Physicochemical properties of LPH produced by chemical and enzymatic processes

**Figure 1:**
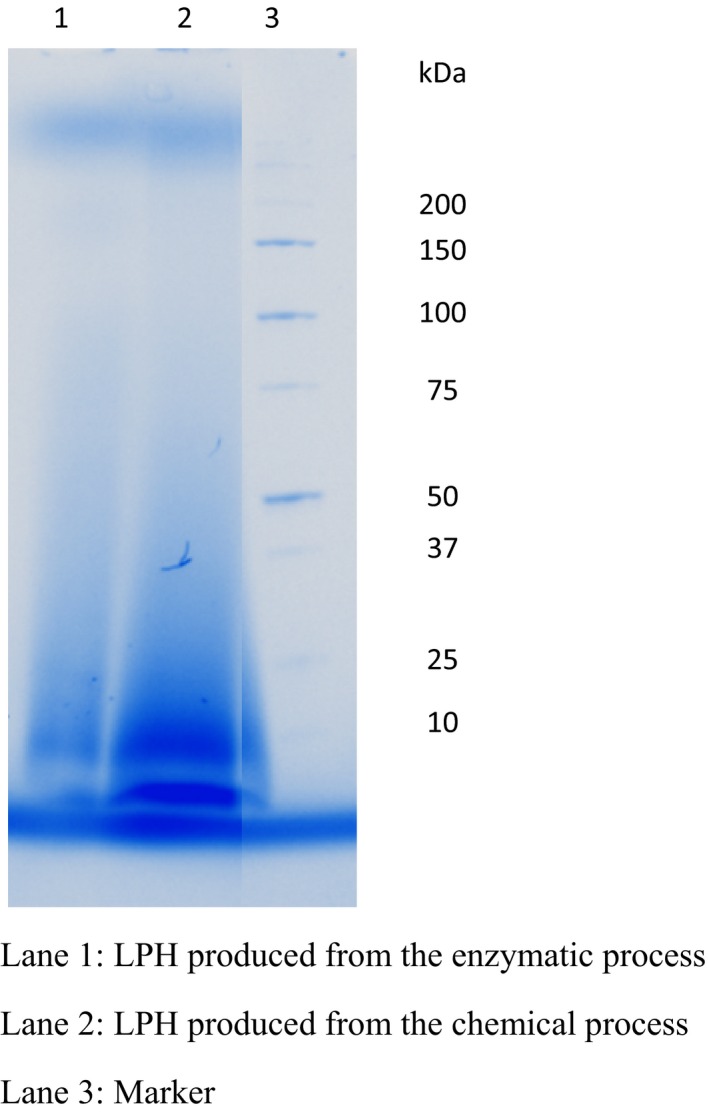
SDS‐PAGE pattern of lobster protein hydrolysates (LPH) produced from the chemical and enzymatic processes.

The oil‐binding capacity and emulsifying capacity, two major physicochemical properties, of LPH produced from chemical and enzymatic processes are presented in Table 3. The oil‐binding capacity of LPH produced from both the chemical (6.32 g oil/g LPH) and enzymatic process (5.98 g oil/g LPH) were significantly lower than the oil‐binding capacity of egg white powder (8.26 g oil/g hydrolysates), the commercial oil binder of food industry. It is therefore not realistic to consider LPH produced from enzymatic or chemical processes as a commercial food grade oil binder. He et al. (2012) addressed that the oil‐binding capacity of protein hydrolysates is associated with the molecular weight distribution of protein hydrolysates. They separated fish protein hydrolysates into five fractions with different molecular weights (>100 kDa, 100–50 kDa, 50–30 kDa, 30–10 kDa, <10 kDa), using membrane fractionation, and found that the fraction with the molecular weight <10 kDa possesses the lowest oil‐binding capacity of 4.45 g oil/g hydrolysates. It can be seen from Figure 1 that the molecular weight of the majority of LPH produced from both the enzymatic and chemical processes is under 10 kDa. This may explain the low oil‐binding capacity of LPH produced from both processes.

**Table 3:** Physicochemical properties of lobster protein hydrolysates produced from the chemical and enzymatic processes

	Oil‐binding capacity^a^ (g oil/g LPH)	Emulsifying capacity^a^ (m^2^/g LPH)
Lobster protein hydrolysates (LPH) produced by the chemical process	6.3^a ^± 0.63	69.7^a ^± 3.69
Lobster protein hydrolysates produced by the enzymatic process	6.0^b ^± 0.95	20.7^b ^± 1.65
Egg white (reference of oil‐binding capacity)	8.3^c^ ± 0.22	
Cow gelatine (reference of emulsifying capacity)		50.3^c ^± 0.61

a Average of the reading of three samples per trial ± standard deviation of the mean. Value within the same column bearing different letters are significantly different at *P* < 0.05, letters do not apply to any row.

However, compared with the reference's emulsifying capacity (50.30 m^2^/g), the LPH produced from the chemical process (69.67 m^2^/g) exceeded this value, though the LPH produced from the enzymatic process (20.69 m^2^/g) is significantly lower. As with oil‐binding capacity, the emulsifying capacity is also associated with the molecular weight distribution of protein hydrolysates. He et al. (2012) discovered that protein hydrolysates with molecular weights lower than <10 kDa have the lowest emulsifying capacity (19.01 m^2^/g), among the five fractions of protein hydrolysates with different molecular weight distributions (>100 kDa, 100–50 kDa, 50–30 kDa, 30–10 kDa, <10 kDa). Figure 1 shows that the molecular weight of the majority of LPH produced from the enzymatic process is below 10 kDa. Therefore, it can be understood why emulsifying capacity of LPH produced from the enzymatic process is low. However, though the molecular weight of the majority of LPH produced from the chemical process is also below 10 kDa, the emulsifying capacity of these protein hydrolysates is significantly higher than that of LPH produced from the enzymatic process. It is also higher than the emulsifying capacity of the reference, the cow gelatine. This result shows that LPH produced from the chemical process may be considered as a commercial food grade emulsifier. The sensory tests, especially bitterness of the protein hydrolysates may need to be done for practical application. However, the standard application of emulsifier in food formulation is about 1–2%, some studies reported that such protein hydrolysates do not have the unwanted flavor at such a low ratio (Dickinson et al. 1988).

Considering that the LPH produced from chemical process acquired significant higher protein recovery (Table 2), higher emulsifying capacity (Table 3) and lower processing cost, in comparison with the LPH produced from enzymatic process, it is fair to conclude that chemical process has the preference over enzymatic process for the production of LPH as emulsifier in food industry.

It is been reported that besides molecular weight, other factors could also affect the emulsifying capacity of protein hydrolysates, including the high solubility, the content of soluble aggregations, and the surface tension (Dickinson et al. 1988). Protein recovery positively relates to the solubility of protein hydrolysates (Wasswa et al. 2007). Therefore, the solubility of protein hydrolysates produced using the chemical process was higher than the solubility of protein hydrolysates produced using the enzymatic process. Similar results have been reported by Vogel (2013), who found that modified soy protein produced using the chemical process improved emulsifying capacity, due to the improved solubility. Aside from the high solubility, it has been reported that the chemical reaction improved the emulsifying capacity of protein‐based products on many occasions, such as on rice protein (Bera and Mukherjee 1989) and carp protein (Fujiwara et al. 1998). This is because of the Maillard reaction between protein and polysaccharide caused by chemical reaction. The chemical reaction forms the complex, which is composed by protein head and polysaccharide strings surrounding the protein centre. Polysaccharide strings are hydrophobic, protein centre is hydrophilic. Polysaccharide strings tend to attract the oil droplet around the water molecular, attracted by protein head. This action creates the emulsifying format of water‐in‐oil, therefore, significantly increased the emulsifying capacity of the original protein‐based products (Zhao et al. 2013). This is the function that single protein molecular, even the commercial emulsifier of cow gelatine, cannot achieve, without chemical reaction.

Using SDS‐PAGE, HPLC and dynamic light scattering, Vogel (2013) found that the improved emulsifying capacity was caused by cross‐linking among protein molecules, the generation of many soluble aggregates and the decrease in surface tension of the soy protein modified by the chemical process.

### Amino acid profile of LPH produced from the chemical process

The results above demonstrate that the chemical process is able to produce LPH with a high production yield and market‐acceptable emulsifying capacity. The amino acid profile of LPH produced from the chemical process was further tested in order to determine the content of essential amino acids, an important parameter to judge the quality of protein‐related food, because essential amino acids cannot be synthesized by human beings, and must be supplied in their diet (Soottawat and Michael 1997).

Table 4 shows the amino acid profile of LPH produced from the chemical process. 30.3% of the amino acids are composed of essential amino acids. This figure is compared with fish protein, the protein that is commonly recognized as a good resource for essential amino acid supplements for human beings. Protein from different parts of Atlantic salmon and Yellowtail kingfish contains essential amino acids from 26% to 36% (He et al. 2011), the same result was also found in fish protein from Arrowtooth flounder and Herring (Sathivel et al. 2004). Therefore, the essential amino acid content of LPH produced from the chemical process is comparable with fish protein. In light of this, it can be concluded that LPH produced from the chemical process can also be used for essential amino acid supplements. Chemical process can lead to unwanted flavor, especially bitterness, in the produced protein hydrolysates. One standard process of flavor masking can be applied to cover the unwanted flavor of these protein hydrolysates (Fujiwara et al. 1998).

**Table 4:** Amino acid profile of lobster protein hydrolysates produced from the chemical process (% of total amino acids)

Amino acid	Lobster protein hydrolysates produced from the chemical process (% of total amino acids)
Alanine	6.9
Arginine	6.5
Aspartic Acid	9.6
Glutamic Acid	15.6
Glycine	8.3
Histidine^a^	2.5
Isoleucine^a^	4.5
Leucine^a^	6.0
Lysine^a^	4.0
Methionine^a^	1.7
Phenylalanine^a^	5.3
Proline	7.5
Serine	6.0
Threonine^a^	5.4
Tyrosine	3.9
Valine^a^	6.3
Total essential amino acids	30.3

a Essential amino acid.

### Heavy metal content of dried RLH and LPH produced from the chemical process

Food safety is a primary concern of any food ingredient. The adverse effects of chronic exposure to heavy metals, especially As and Hg, have been the subject of long‐standing concerns, due to their association with skin cancer and organ cancers (Jonathan and Dean 2007). There are two types of As: inorganic As and organic As. Only inorganic As is the form that tends to be more toxic and has been linked to cancer (Kevin 2010). Besides As, Hg also has important impact on the quality of seafood because it is a heavy metal commonly existing in marine environment. Choi et al. (2011)reported that intake and potential health risk of butyltin compounds from seafood consumption positively associated with the high content of Hg in seafood. In light of this, the content of heavy metals, especially inorganic As and Hg, has been considered a priority for the assessment of seafood safety.

The heavy metal content of dried RLH and LPH produced from the chemical process is shown in Table 5. Table 5 shows the regulatory standards according to Australia New Zealand Food Standard (FSANZ) 1.4.1. The results demonstrate that the contents of all heavy metals, including inorganic As, were lower than the regulatory standard levels, regardless of whether it was raw material or processed LPH. Therefore, the LPH produced from the chemical process can be applied safely in food formulations.

**Table 5:** Heavy metal content of dried rock lobster head and lobster protein hydrolysates produced from the chemical process

Heavy metals	Standards (mg/kg) regulated by FSANZ	Raw materials	Produced products
		Dried rock lobster head (mg/kg)	Lobster protein hydrolysates (mg/kg)
Inorganic As	2	0.2	0.18
Cd	2	0.1	<0.01
Pb	0.5	<0.01	0.21
Hg	0.5	<0.01	0.16
Sn	250	<0.01	18

## Conclusions

Rock lobster heads are currently disposed of without any value‐added utilization, but a cost to the industry. This study has developed and compared an enzymatic and a chemical process to produce LPH as a functional food ingredient from RLH. It was found that the chemical process is more cost‐effective, due to its 30% higher LPH production yield. In addition, the chemical process also produced LPH with an emulsifying capacity that exceeded the emulsifying capacity of cow gelatine, the commercial food grade emulsifier. Furthermore, this study also found that LPH produced from the chemical process can be used as essential amino acid supplements, due to their high content of essential amino acids. All the contents of heavy metals, including inorganic arsenic, of the LPH produced from the chemical process were lower than the regulatory standard levels. In conclusion, the lobster protein hydrolysates produced from RLH by the chemical process are preferred and safe to be applied in food formulations, which open a new avenue for value‐added to rock lobster processing waste to improve the profitability and sustainability of Australian rock lobster industry.

## Conflict of Interest

None declared.
